# MALIGNANT MELANOMA – CUTANEOUS METASTASES

**DOI:** 10.4103/0019-5154.44803

**Published:** 2008

**Authors:** L Padmavathy, L Lakshmana Rao, N Ethirajan, B Krishna Swamy

**Affiliations:** *From the Division of Community Medicine, Rajah Muthiah Medical College, Annamalai University, Annamalai Nagar - 608 002, Tamil Nadu, India*; 1 *From the Division of Pathology, Rajah Muthiah Medical College, Annamalai University, Annamalai Nagar - 608 002, Tamil Nadu, India*

**Keywords:** *Cutaneous metastases*, *melanoma*, *maignant*

## Abstract

Melanoma composed of melanocytes may arise in the skin or other tissues harboring melanocytes, such muco-cutaneous junctions, mucosa including the conjunctiva, iris, choroids and substantia nigra.^1^ Metastases to the skin and subcutaneous tissues from a malignant melanoma are less common. A case of multiple painless nodules on the body that revealed metastatic deposits of melanoma on histopathological examination is being reported.

## Introduction

Melanoma composed of melanocytes may arise in the skin or other tissues harboring Melanocytes, like muco -cutaneous junctions, mucosa including the conjunctiva, iris, choroids and substantia nigra.^1^ Metastases to the skin and subcutaneous tissues from a malignant melanoma are less common.

Cutaneous metastases of internal malignancies are relatively uncommon. Autopsy studies done on patients with internal malignancy have recorded 0.7%–9% incidence.^2^ Most cases occur late in the course of the disease, but cutaneous metastases may be the initial presentation of the internal malignancy.

## Case Report

A 70-year-old male presented with multiple, painless skin-colored nodules on body of one month duration.

**Fig. 1:**
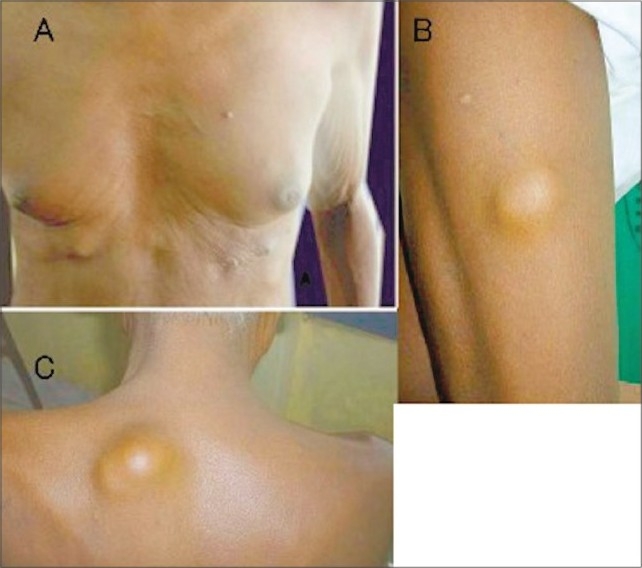
A. Multiple nodules over the body

The nodular lesions were distributed on the front and back of chest and right thigh. The lesions were not tender, firm to hard in consistency, varying in size from 2 to 5 cm, fixed to the skin and the underlying tissues (Fig. 1). He had vitiligo of the oral mucous membrane and soles. He is a known diabetic on irregular treatment. He gave a history of an ulcer on the right sole between the 2nd and 3rd toes 2 years earlier following a thorn prick, for which he received some surgical treatment and the details of which were not available.

**Fig. 2:**
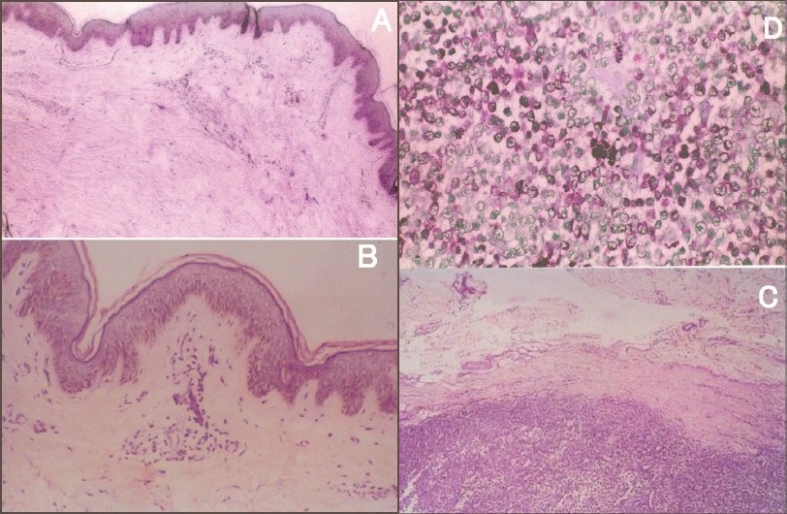
Skin biopsy from nodule – showing epidermis and subepithelial stroma with sparse mononuclear cell collections. Note that the stratified squamous epithelium is unremarkable, × 10 H and E Stain (A) and × 20 H and E stain (B). A circumscribed nodule composed of round to oval cells with prominent nucleoli, × 20 H and E (C). Tumor nodule showing prominent nucleoli and pigment, × 40 H and E (D).

Systemic examination did not reveal any abnormality. There was no lymphadenopathy. His diabetes was not under control, with fasting blood sugar (FBS) at 201 mg% and post prandial blood sugar (PPBS) at 257 mg%. Other hematological and biochemical investigations were within normal limits. Ultra sonogram of the abdomen and X-ray chest also were normal. An fine needle aspiration cytology from a nodule on the back and an excision biopsy from a nodule in the infra mammary region revealed the skin and subcutaneous tissues with a few collections of mononuclear cells in the dermis without any junctional activity. A well circumscribed nodular lesion composed of melanocytes with prominent nucleus and nucleoli and condensed chromatin arranged in tight nests in the dermis. No continuity could be traced between the nodular lesion and the overlying skin (Fig. 2).

## Discussion

The etiology and pathogenesis of cutaneous melanoma is not known. Epidemiological studies demonstrate a role for genetic predisposition and sun exposure in melanoma development. The major gene involved in melanoma development resides on chromosome 9p21. This gene known as CDKN2A encodes two separate gene products that are negative regulators of cell cycle progression.^3^

Melanoma composed of melanocytes may arise in the skin or other tissues harboring Melanocytes, like muco-cutaneous junctions, mucosa including the conjunctiva, iris, choroids and substantia nigra.^1^

Acral lentiginous melanoma is the most common form of melanoma in dark-skinned people and is essentially limited to the palms, soles and subungual regions^4^ and is an aggressive tumour.^5^

Since exact details are unavailable, it could be surmised that our patient had an acral lentiginous melanoma on the right sole earlier and has presented with metastatic deposits now.

Melanomas spread via lymphatics or by hematogenous dissemination.^5^ Metastatic or stage IV malignant melanoma is a devastating disease.^6^ It is defined by dissemination of the cutaneous tumor to other organs or nonregional lymph nodes.

Metastasis occurs in 15%–26% of stage I and stage II melanoma. The spread of disease from the primary site usually occurs in a step wise sequence: primary melanoma → regional metastasis → distant metastasis. However, distant metastasis can occur skipping the regional lymph nodes and indicate hematogenous spread^3^ as could be presumed to have happened in our patient.

The skin, subcutaneous tissues and lymph nodes are the first site of metastatic disease in 59% of patients.^6^ Hematogenous dissemination can give rise to widespread metastasis as was observed in our patient, although there was neither hepatomegaly nor pulmonary metastases. Metastases to the skin and subcutaneous tissue were reported in 54%–75% of patients on autopsy.^7^

The distinguishing points between melanoma metastases in the skin and the primary tumor are absence of an inflammatory infiltrate and the junctional activity (which is proved by a negative DOPA reaction) in the former.^8^

In about 4% of patients with metastases, no primary tumor could be found. The present case on histopathological examinations was diagnosed as metastases of melanoma and search for a primary tumor was advised. The primary tumor could be in an internal organ or might have regressed spontaneously. In the present case, in view of no definitive primary lesion, the possibility of spontaneous regression could also be entertained.

The most specific and sensitive test to detect metastatic disease are the melanoma-associated antigen protein S100, melanoma inhibiting activity (MIA), and the melanin precursors 5-S-cysteinyl-DOPA and the ratio of L-DOPA/L-Tyrosine. The detection of circulating melanoma cells in blood is a potential surrogate marker for subclinical residual disease. Tyrosine's mRNA remains the best target for the detection of circulating metastatic melanoma cells by real time polymerase chain reaction (RT-PCR).^8^ However, due to lack of facilities, none of the above investigations could be undertaken.

The median survival is approximately 7 months for all patients with metastatic disease. Moreover, survival is better with a longer duration of remission after primary disease, fewer metastatic sites involved and in those with nonvisceral disease.^6^

Treatment for metastatic disease remains unsatisfactory. Many drugs have been used, such as dacarbazine, timozolamide, vinca alkaloids, nitrosureas, tamoxifen and immunotherapy, with varying results.^6^

Death due to metastases of cutaneous melanoma is wholly preventable by completely excising the macules and patches at an early stage. Metastatic nodules signify a poor prognosis. In view of the numerous lesions in our patient, surgery was not contemplated and he was lost for follow up.
